# Involving users in the design of a randomised controlled trial of an intervention to promote early presentation in breast cancer: qualitative study

**DOI:** 10.1186/1471-2288-10-110

**Published:** 2010-12-22

**Authors:** Lindsay JL Forbes, Carol McNaughton Nicholls, Louise Linsell, Jenny Graham, Charlotte Tompkins, Amanda J Ramirez

**Affiliations:** 1 King's College London Promoting Early Presentation Group, St Thomas' Hospital, London SE1 7EH, UK; 2 National Centre for Social Research, 35 Northampton Square, London, EC1V 0AX, UK

## Abstract

**Background:**

The purpose of this study was to explore women's views of the design of a large pragmatic cost-effectiveness randomised controlled trial of the policy of offering a health professional-delivered intervention to promote early presentation with breast symptoms in older women and thereby improve survival, with a view to informing protocol development. The trial will recruit over 100,000 healthy women aged 67+, and outcome data will be collected on those who develop breast cancer. The scale of the trial and the need for long-term follow-up presented a number of design challenges in relation to obtaining consent, ascertaining and contacting participants who developed breast cancer, and collecting outcome data.

**Methods:**

Qualitative study involving 69 women participating in 7 focus groups and 17 in-depth interviews. 15 women had a previous diagnosis of breast cancer and 54 did not.

**Results:**

The women held strong views and had a good understanding of the rationale of the design of clinical trials. The women recognised that in a very large trial with long-term follow-up it was necessary to incorporate design features to make the trial feasible and efficient. Most strikingly, they supported the idea of opt-out consent and identifying women with breast cancer using routine datasets.

**Conclusions:**

This model of user involvement engaged women well with the design challenges of the trial and led to improvements to the protocol. The study strengthens the case for user involvement, in particular through focus groups and in-depth interviews, in the design of trials.

## Background

It is widely accepted that involving service users in the design of research makes for more relevant research questions, higher levels of participation, better study design, and better interpretation of findings [1]. The Department of Health Research Governance Framework recommends that "relevant service users..... should be involved wherever possible in the design, conduct, analysis and reporting of research" [2]. However, triallists rarely report whether users have been involved in the research and where they have, what happened as a result [3], which makes it difficult to judge how useful the user involvement was.

We have designed a randomised controlled trial to evaluate the policy of offering a complex low-risk intervention to promote early symptomatic presentation of breast cancer in older women, and thereby improve survival. The intervention, known as the Promoting Early Presentation (PEP) Intervention, consists of a 10-minute interaction with a health professional supported by a booklet. It is designed to provide older women with information about the symptoms and risk of developing breast cancer, and the motivation, confidence and skills to present promptly on discovering a breast change [4,5]. The rationale is that a high proportion of older women delay presentation with breast cancer [6] and that this delay is associated with worse survival [7]. We have already shown in a randomised controlled trial of 867 women that the intervention increases breast cancer awareness at one year compared with usual care [8]. The hope is that the increased breast cancer awareness (which encompasses motivation, confidence and skills as well as knowledge) will lead to earlier presentation and thereby improve survival.

We are now planning a much more ambitious cluster randomised controlled trial which will examine whether the policy of offering the PEP Intervention following final routine mammogram on the National Health Service (NHS) Breast Screening Programme (at the age of 67-73) promotes prompt presentation and improves survival compared with not offering the PEP Intervention. The trial will need to recruit tens of thousands of women over the whole of England because only a small proportion of participants will develop breast cancer (and, therefore, provide outcome data on duration of symptoms and breast cancer survival) over the follow-up period of five to eight years. We have chosen a cluster design in this pragmatic trial to avoid the disruption to services caused by individual randomisation in clinic, and because there will be lower risk of missing potential participants and of contamination. In addition, the PEP Intervention must be delivered by a specially trained radiographer; with individual randomisation, such a radiographer would have to be present at every screening clinic, rather than simply at those in clusters randomised to offer the PEP Intervention.

We incorporated a number of features into the design of the trial to ensure that it would be feasible. These related to the consent model and the methods of ascertaining, contacting and interviewing participants with breast cancer.

### Consent model

Individual trial participants can, potentially, give consent to randomisation, participation and follow-up. Consent from individuals to be randomised as a part of a cluster in a trial is rarely possible [9,10]. Consent to participate and be followed up are, on the other hand, possible and desirable in a cluster-individual trial such as ours [11], where individuals within clusters may be offered an intervention (in contrast to a cluster-cluster trial where the intervention may be applied to the whole cluster with no real option for an individual to opt out, for example, water fluoridation).

In our trial, because of the low risk associated with participation, we proposed that consent to participate and be followed up, in both control and intervention arms, should be obtained using "opt-out" consent, rather than the more traditional written opt-in consent. We planned to seek this consent at the time of postal invitation to participate sent with the invitation for a screening appointment, from women randomised both the control and intervention arms. All participating women, in both control and intervention arms would be provided with full written information about the trial, including the contact details of a researcher with whom to discuss participating if they wished, and would be able to opt out of participating or being followed up by post, telephone, online or in person when attending for breast screening. If they opted out, they would remain a member of relevant cluster (to be offered either the PEP Intervention or not), but no data would be collected about them and they would not be offered the PEP Intervention should they be randomised to a cluster in the PEP Intervention arm. We planned to obtain a further level of informed consent from women in intervention clusters to receive the PEP Intervention on arrival at the screening appointment: this would be opt-in verbal consent (providing the woman had not opted out earlier).

### Methods of ascertaining, contacting and interviewing participants with breast cancer

We proposed to identify trial participants who developed breast cancer by flagging their NHS numbers on routine datasets, a method commonly used in trials to identify deaths among participants [12]. This would be minimally intrusive and would, therefore, also, minimise cost. We proposed then to ask the women's breast care teams to seek consent from the women to be contacted by our research team with a view to conducting an interview to collect key outcome data on nature and duration of symptoms. The reason for asking the breast care team to coordinate consent for interviews is that the clinicians would be able to judge the appropriate timing of the interview and identify whether it might have negative consequences for the woman - it is possible that the interview could cause distress, for example, by resurrecting the fear and anxiety at the time of symptom discovery or diagnosis or regret at having delayed presentation.

We proposed to conduct interviews (having obtained written opt-in consent), over the telephone rather than face-to-face, at about eight to ten weeks after diagnosis. These interviews would collect key outcome data: nature and duration of symptoms. Other key outcome data in the trial would be collected from cancer registry data, for example, date of diagnosis and stage.

We wished to ensure that these proposed methods were not considered a threat to the dignity, safety, rights or well-being of the trial participants. We carried out a qualitative study using focus groups and in-depth interviews to examine women's perceptions of these elements of trial design.

## Methods

We carried out focus groups and in-depth interviews with women aged 60 to 75, some who had been previously diagnosed with breast cancer and others who had not had breast cancer. We recruited women with breast cancer with the help of two cancer charities: Macmillan, which advertised for volunteers on its website, and Breast Cancer Care, which sent out a flyer with its newsletter. We also directly contacted a Asian women's breast cancer support group. We recruited women who had not had a previous diagnosis of breast cancer using research recruitment agencies, who approached women in public areas and from existing databases of people willing to take part in research, to invite them to participate. We aimed to achieve a mix of women by age and income and living in rural and urban locations.

During each focus group and interview, the facilitator explained the trial design issues and used a topic guide. In the focus groups, the facilitator supplemented this with a vignette describing one imaginary trial participant's experience of taking part in the trial ('Barbara's story'). All focus groups and interviews were audio-recorded. We paid all participants £20 plus travel expenses.

Interviews and focus groups were transcribed verbatim and the data managed and analysed using the Framework method [13]. This involved developing a thematic framework, whereby columns represented themes and rows represented an individual participant or groups of participants. We summarised data from each participant and/or group in the relevant cell (retaining the page of the transcript from which it was taken, so that it was possible to return to a transcript to explore a point in more detail). We then identified patterns, explanations and associations from the data.

The National Centre for Social Research Ethics Committee gave ethics approval for this qualitative study. All participants provided verbal consent to take part.

## Results

We carried out seven focus groups and seventeen in-depth interviews in total. Table 1 shows the demographic characteristics of the women included in the study. We achieved a good range of characteristics by age, ethnic group, income, rural or urban residence and living arrangements.

**Table 1:** Characteristics of participants

Characteristic		Previous diagnosis of breast cancer		
		**Yes**	**No**	**Total**

Age	60 - 65	10	20	30
	66 - 70	2	13	15
	71 - 75	3	20	23
	Unspecified	0	1	1

Ethnic group	White British	14	36	50
	Non-white	0	18	18
	White other	1	0	1
	Unspecified	0	0	0

Household income	Under £20,000	6	20	26
	£21,000 - £39,000	3	14	17
	£40,000 and above	4	7	11
	Unspecified	2	13*	15

Living arrangements	Lives alone	6	15	21
	Lives with partner	8	24	32
	Lives with others	1	5	6
	Unspecified	0	10*	10

Area	London	5	36	41
	Outside London	10	18	28

*These data were not collected from the ten women participating in the Bengali focus group.

We carried out two focus groups and nine in-depth interviews with women previously diagnosed with breast cancer (total 15 participants). The focus groups were held with two established groups of women, one based in London and one in a market town in the Midlands: a support group, and a volunteer group providing support for women with breast cancer at a hospital. All these women were of white British ethnic origin.

We carried out five focus groups (two in rural locations and three in London) and eight in-depth interviews with women who had not had breast cancer (total 54 participants). One of these focus groups was made up of Bengali-speaking women. Four of the in-depth interviews with women without breast cancer were with Black British women.

The focus groups were held in private rooms in community centres and restaurants. The in-depth interviews took place at the participants' homes, except for two with women diagnosed with breast cancer, which were carried out over the telephone.

### Consent model

#### Initial opt-out consent

The women (irrespective of experience of breast cancer, ethnicity or location) expressed support for the opt-out model for initial consent to participate and to be followed up using routine datasets. The women expressed understanding the need for trials to recruit a high proportion of potential participants, that recruitment should not be biased, and that trials had to be cost-effective; they felt that opt-out consent would help to achieve this. They also felt that opt-out consent would be much easier for potential participants, and was fair and inclusive. They thought that it might reduce bias in recruitment: because women might otherwise be inadvertently excluded simply because it was not convenient at the time or easy to complete an opt-in form, rather than because they really wished not to take part.

*"I like the idea of opting out so then I'm included and I won't feel then left out or have to chase anybody up to be in it." *(Focus group participant, No breast cancer)

The women felt that the only reason they might wish to opt out was because of the time commitment involved in taking part, as they felt taking part in the trial was not likely to be painful or intrusive.

Participants thought that it was sufficient to inform women about the follow-up using routine datasets and that seeking formal consent for this aspect of participation was unnecessary.

The idea of opt-out consent to being recontacted in the event of developing breast cancer generated a more mixed response. The women who had not had breast cancer felt unsure about how they might feel about this if they developed breast cancer. Some realised that even if women opted out of being recontacted at recruitment to the trial, they might later change their mind. The general view across the participants (regardless of characteristics) was that any kind of consent (opt-in or opt-out) for future contact at the time of recruitment may not be valid several years later under different circumstances.

*"Well, to be honest, until you've had cancer, you don't know, you can't imagine how you feel. So you might quite happily tick a box and say, 'Yes if I get cancer I'll talk to you', but again that might be a different kettle of fish if you develop cancer. So I don't think you know yourself how you would cope and how you would feel." *(Interview participant 1, with breast cancer)

#### Opt-in verbal consent to receive the intervention

The women supported the idea of verbal consent to receive the intervention. While comfortable with providing written consent, they expressed the view that the main advantage of written over verbal consent was to protect the researchers or the NHS, not the participants. They said that written consent could be perceived as too formal, which might inhibit people from taking part.

*"I think I'd be quite happy just to be of any use or any help, you know, in any way at all. And I wouldn't feel that I had to sign a piece of paper to say yes I will do this. That's almost being - I think signing things is almost a bit more beholden." *(Interview participant 2, with breast cancer)

They felt in that in a trial of this kind of intervention written consent was not necessary, because the potential for harm was low and that the participant would be capable of making an informed decision to receive the intervention or not.

The women recognised that written consent would generate paperwork and this would incur costs and storage issues.

*"[written consent is] not really necessary is it? I wouldn't have thought so. I think verbal is fine. I really do. I mean we've got enough paperwork anyway. I know it doesn't sound much but if you're doing thousands and thousands of women there's a cost to that you know, and I don't see the point of it. I really don't." *(Interview participant 3, with breast cancer)

The Bengali group noted that verbal consent might be more valid for people who did not have a good command of written English, including those with a low level of literacy in any language. In addition, they would be able to provide consent without a family member, or other, reading out written material to them, thus promoting their confidentiality.

#### Informed consent

Several participants noted that consent to take part in research, however it was given or recorded, would only be valid if the participant was fully informed about the implications of taking part. Indeed ensuring that respondents felt informed was felt to be of greater significance than the mode of consent gained - verbal or written.

*"It is important... just to keep people informed. In most cases... if you're not kept informed is when people get cross and angry about things, 'Well nobody has told me this'..." *(Interview participant 4, with breast cancer)

### Identification of women with breast cancer through routine datasets

Participants recognised the need to identify women with breast cancer through routine datasets in the context of this trial and were generally comfortable with the idea. There was some expectation, especially among the women with breast cancer, that this was routinely done.

Participants felt that women should be informed in clear language about the intention to follow them up through routine datasets and the reasons for doing so. Some expressed the view that it was important to outline specifically the plans for using the data.

*"It has to be made clear in the beginning that if you take part in this, we will use your date of birth and your NHS number. We will then track you this year and this year." *(Interview participant 4, with breast cancer)

Participants had heard about losses of identifiable electronic data held by the government. Some were concerned that the routine datasets might not be stored securely during the research but felt less concerned knowing that the datasets would not contain names and addresses, only NHS numbers. They felt that the risks of retaining data were appropriately balanced against the importance of the research. They felt the need for assurances that the data would be used only for the purposes of the trial, that access would be restricted to appropriate research staff trained to handle and analyse the data and that the data would not be available to pharmaceutical or insurance companies.

### Recontact to take part in an interview

The participants acknowledged that taking part in the interview to collect data on the nature and duration of symptom had the potential to lead to some negative emotions. They generally approved of the idea that the breast care team (in particular, a breast care nurse) should coordinate making contact with a woman with breast cancer to ask them to participate in an interview to collect outcome data This was because the nurse would be known and trusted, and could advise the researchers about the appropriateness of contact at a particular time. It was considered important that the breast care team provided clear and concise information about the nature of the interview to allow women to make an informed decision about taking part.

Participants felt that women would be comfortable declining to be contacted by the researchers and would not feel an inappropriate level of obligation or pressure to take part.

Women with breast cancer identified some disadvantages of contact being coordinated by the breast care team. Some had not experienced a high level of continuity of care from their breast care teams, and thought that women with similar experiences might find this approach unacceptable.

Among the women with experience of breast cancer, views about the proposed timing of recontact - eight to ten weeks after diagnosis - varied. Some felt that this timing was appropriate because women would probably have recovered from the initial distress surrounding diagnosis and that the interview might provide a welcome distraction during treatment. However, some felt that this was too early because women might still be having treatment or recovering. None felt that contact should be made earlier than eight weeks after diagnosis. Participants thought that the women should be able to choose the timing of the interview.

Several participants thought that data on time from symptom discovery to presentation should be collected face-to-face rather than by a telephone interview. Some participants thought that telephone interviewing would be unpleasantly impersonal, but others suggested possible advantages of telephone interviewing: it might be less embarrassing, less threatening, less time-consuming, cheaper and more convenient if the woman was busy, ill or living in a rural area.

## Discussion

This qualitative study including focus groups and in-depth interviews in women with and without personal experience of breast cancer found broad support for an unconventional consent model, a process of identifying women with breast cancer using routine datasets and a proposed method of recontact to take part in an interview. We found limited support for the idea of collecting data on nature and duration of breast cancer symptoms using a telephone rather than a face-to-face interview.

The results provided many insights leading to improvements to the protocol and the information provided for participants. In particular, we gained support for our proposal to seek opt-out consent for randomisation or follow-up using routine data; women did not see this as a threat to their rights, safety or well-being. However, seeking opt-out consent to be recontacted on developing breast cancer was not considered sufficient; in fact, some expressed the view that consent of any kind at recruitment to be recontacted some time later under very different circumstances, in other words, in the event of the woman developing breast cancer, might not be valid, because she might feel very different about it at that time - more positively or negatively disposed towards it. We also gained support for the idea of verbal consent to receive the intervention, once opt-out consent to participate had been given.

In relation to follow-up using routine data, the results suggested that not only did we need to develop robust systems for data governance but also to reassure participants about these systems. It also suggested that we should accommodate women's choices about when to be interviewed about nature and duration of symptoms in the event of developing breast cancer. The study suggested that face-to-face interviews with women to collect data on the nature and duration of symptoms might be seen to respect the well-being of participants more effectively than telephone interviews.

We were surprised by the support for initial opt-out consent to participate; we had expected participants to think that this might lead to some women being included against their will due to inertia or simply not reading the postal materials. No participants expressed this view: they felt that opt-out consent was inclusive and cost-effective, and that it would help maximise participation and reduce bias in recruitment. Their responses suggested that they thought that these considerations overrode the need for detailed opt-in consent. This may be because the participants recognised that the participants would not be asked to opt out of receiving the PEP Intervention, rather, they would be asked to opt out of being *offered *the PEP Intervention (which they would still be at liberty to decline) and to be followed up using routine data. It is clear that consent to receive any intervention should be obtained actively, using opt-in methods (although not necessarily recorded in writing) [14][15].

Opt-in consent may be seen as more defensible ethically and legally than opt-out consent as it relies on a participant actively confirming that they have consented (often by ticking or initialling a box and signing a form) rather than solely omitting to withdraw consent. Opt-out consent may be seen as a threat to participant autonomy, a fundamental principle of medical ethics, because failure to withdraw consent does not confirm that the participant has actively consented. An opt-out consent model has, however, been used with NHS research ethics committee approval in trials of low risk interventions [16]. Opt-out consent increases recruitment and leads to less biased samples of participants and has been recommended as the consent model of choice for trials of this kind [17]. It is important in our trial that we ensure that participants are provided with the option of opting out at any time after initial recruitment.

A striking finding was the idea that written opt-in consent was not always desirable and that verbal opt-in consent might be preferable. The participants thought that written opt-in consent was too formal, and that it might inhibit women from taking part or make them feel beholden to the researchers. They also felt that that it protected the researchers or the NHS, not research participants. The interpretation of written opt-in consent as a contract or a liability waiver has been noted before [18]. Another disadvantage of written consent rather than verbal consent expressed was that it was less likely to be valid in people of limited literacy or command of written English.

The guidance provided to National Health Service Research Ethics Committees emphasises the importance of achieving *informed *consent to take part in research, rather than focusing on whether it is written or verbal, or whether it is opt-in or opt-out http://www.nres.npsa.nhs.uk/applications/guidance/consent-guidance-and-forms/. Research ethics committee also refer to the Department of Health guidance on consent to receive physical interventions [15] (which the PEP Intervention could, arguably, be seen to be, although it consists simply of a one-to-one discussion). This emphasises that health professionals should assess capacity to consent, ensure that consent is given voluntarily and ensure that sufficient information is provided. The guidance is explicit that the validity of consent does not depend on the form in which it is given. It states that written consent 'merely serves as evidence of consent; if the elements of voluntariness, appropriate information and capacity have not been satisfied, a signature on a form will not make the consent valid'. There is no requirement in British law for consent to be written (except for defined procedures such as fertility treatment). Also relevant in the UK is the General Medical Council's guidance on consent [14]. This also focuses on the need to provide full, balanced information. This advises that consent for minor or routine investigations or treatments may be verbal or implied (for example, for phlebotomy or blood pressure measurement). It also advises (although does not require) that doctors obtain consent for treatments that are part of a research programme. Whether the PEP Intervention should be defined as a 'treatment' is not clear.

We were also surprised at the participants' support for the idea of identifying women with breast cancer using routine datasets; we had expected that this would be seen to be a threat to privacy given recent media coverage of losses of governmental electronic datasets including identifiable data. However, this is consistent with other findings [19][20].

It was striking that the participants had a good understanding of the experimental nature of clinical trials. This was in contrast to the findings of a Health Technology Assessment report on the public understanding of randomised controlled trials, which suggested poor public understanding of the rationale for the randomised controlled trial design [21]. The participants in our study recognised the need for trials to be conducted efficiently, and that their primary aim was to answer the research question; women did not find this difficult to accept. This understanding may have been enhanced by the lengthy introductory session to the interviews and focus groups outlining the proposed trial which material such as vignettes to promote participants' comprehension. Such measures could be considered good practice.

The main limitation of our qualitative study may be the representativeness of the participants. That women were willing to participate in research of this kind suggests that they were more educated about health research than average. This may have meant that they were less likely to find some aspects of trial design unpalatable compared with the general female population. Only five of the fifteen participants with breast cancer were aged over 65, which we recognise as a limitation, in view of the fact that the incidence of breast cancer increases with age; this means that we may not have adequately ascertained the views of older women with breast cancer. Also, none of the women with breast cancer belonged to non-white ethnic groups, so we were unable to comment on the views of this group (although these women represent a small proportion of the population of the UK, and have a lower incidence of breast cancer than white women [22]).

The most common forms of user involvement in randomised controlled trials is in the drafting of trial information leaflets and promoting recruitment, and users sometimes sit on steering committees [3]. We have found only a small volume of published evidence showing that users can influence the design of randomised controlled trials. The studies that we found describing concrete changes to trial design as a result of the user involvement used, like ours, focus groups and interviews. In the design of a trial of oxygen supplementation after acute stroke, a quantitative and qualitative study of people with stroke and their carers led to the identification of the most appropriate outcome measures, clarified the follow-up method to be used and influenced the consent model [23]. During the design of a randomised controlled trial of thrombolysis in acute stroke, a qualitative study including focus groups to seek the views of older people led to the design of a potentially controversial consent model, including consent from next of kin [24]. During the design of a trial to evaluate antiretroviral and nutritional interventions to reduce mother-to-child transmission of HIV during breastfeeding in Malawi, an interview and focus group study of potential participants identified unforeseen potential problems with trial implementation - for example, the propensity of study participants to share out antiretroviral medication and food supplements given as part of the trial - and led to identification of ways to minimise their impact [25].

The focus group and in-depth interview model of user involvement requires trained qualitative researchers, a significant amount of work developing materials and are, therefore, expensive. However, for our trial, using this model generated surprising insights to strengthen the protocol, which is, arguably, less likely to occur with more common and conventional models of user involvement such as sitting on steering groups.

Researchers are sometimes negative about user involvement in trials, suggesting that it takes too long, that expectations are too high, and that there is a conflict of interest between the "role of a patient advocate and reliable assessment of cost-effectiveness" [3]. Using carefully facilitated focus groups and in-depth interviews with well-prepared materials, expectations of users were appropriate and that there was no conflict of interest between the role of the user and good study design.

## Conclusions

A model of user involvement in research design, employing focus groups and in-depth interviews, engaged women well with the design challenges of the trial and led to improvements to the protocol. The study strengthens the case for user involvement of this kind in the design of trials.

Opt-out consent to participate in trials is acceptable to potential research participants and should be considered for low risk interventions. Use of routine datasets is also acceptable, given appropriate governance arrangements, and should also be considered in trials where long term follow up for relatively rare events is needed.

## Competing interests

The authors declare that they have no competing interests.

## Authors' contributions

LF, LL and AJR designed the randomised controlled trial and had the idea for the study. LF commissioned the study, contributed to study design and wrote the paper. CMN, JG, and CT contributed to study design, and collected and analysed the data. All authors read and approved the final manuscript.

## Pre-publication history

The pre-publication history for this paper can be accessed here:

http://www.biomedcentral.com/1471-2288/10/110/prepub
